# Examining the Utility of Resective Epilepsy Surgery in Children With Electrical Status Epilepticus in Sleep: Long Term Clinical and Electrophysiological Outcomes

**DOI:** 10.3389/fneur.2019.01397

**Published:** 2020-01-15

**Authors:** Ahmad Marashly, Jennifer Koop, Michelle Loman, Yu-Wen Lee, Sean M. Lew

**Affiliations:** ^1^Division of Pediatric Neurology, Children's Hospital of Wisconsin, Medical College of Wisconsin, Milwaukee, WI, United States; ^2^Division of Pediatric Neuropsychology, Children's Hospital of Wisconsin, Medical College of Wisconsin, Milwaukee, WI, United States; ^3^Division of Pediatric Neurosurgery, Children's Hospital of Wisconsin, Medical College of Wisconsin, Milwaukee, WI, United States

**Keywords:** electrical status epilepticus in sleep, epilepsy surgery, hemispherotomy, seizure outcome, cognitive outcome

## Abstract

**Background:** Electrical Status Epilepticus in Sleep (ESES) is an epileptic encephalopathy syndrome characterized by infrequent clinical seizures and prominent interictal burden during slow wave sleep associated with cognitive deficits and behavioral dysfunction. Medical treatment with anti-epileptic drugs is often unsuccessful. Resective surgery may be a valuable option in carefully selected patients. This case series aims to describe the indications, long term results and utility of resective surgery for ESES.

**Methods:** Information on 14 patients who underwent surgery for epilepsy and ESES at the Children's Hospital of Wisconsin between 2007 and 2017 is included. Clinical, electrographic and neuropsychological features and outcomes are described in detail.

**Results:** The most common pathology was encephalomalacia due to perinatal middle cerebral artery stoke (5/14). Twelve patients had imaging findings of perinatal pathologies; however, two patients had normal magnetic resonance imaging. Surgery was performed to control refractory epilepsy in eight patients. Six patients had no clinical seizures for 1–6 years prior to surgery, one of which had no known clinical seizures at all. All showed cognitive declines (6/14) or impairment (8/14) on neuropsychological assessments, and surgery was suggested to minimize further cognitive declines. The most common surgical procedure was hemispherotomy (10/14). Temporo-parieto-occipital disconnection, frontal lobectomy, parieto-occipital resection, and limited corticectomy were also used, with good outcomes for the first three procedures. Clinical follow up mean was 4.4 years and 12 patients had excellent seizure outcome. Electroencephalography (EEG) follow up mean was 3 years and ESES resolved in 12/14 patients. All patients completed post-surgical neuropsychological evaluation with mean follow-up of 17.46 months.

**Conclusions:** Resective surgery is an effective treatment for selected cases of ESES, producing long term seizure freedom, resolution of ESES and stabilization of cognitive and behavioral functioning in most patients. Our case series is the largest single center cohort description addressing resective surgery for ESES. Outcomes in this sample suggest that good long-term seizure, EEG and cognitive/behavioral outcomes can be achieved in patients with normal brain imaging and in limited lobar or multi-lobar resections. Moreover, patients with ESES and very infrequent clinical seizures can benefit from surgery with stabilization of cognitive and behavioral functioning.

## Introduction

Electrical Status Epilepticus in Sleep (ESES) is an epileptic encephalopathy syndrome characterized by very frequent or near continuous interictal epileptiform discharges during non-Rapid Eye Movement sleep (NREM); infrequent or rare clinical seizures; and variable neurocognitive deficits, it was first described in 1971 (1). The International League Against Epilepsy (ILAE) introduced the term epileptic encephalopathy with Continuous Spike and Waves during Slow Sleep (CSWS) in 1989 to encompass the clinical and electrographic aspects of this epileptic phenomenon (2). Although the term ESES was initially coined to described the electrographic findings only, ESES and CSWS have been used interchangeably over the years referring to both the electrographic and clinical features (3). In the current paper, ESES is used to describe both the clinical and electrographic features of this epileptic encephalopathy.

Variable etiologies can lead to the development of ESES including congenital, acquired, focal and generalized pathologies, such as malformations of cortical development (4), hydrocephalus (5), and evolution from benign focal epilepsies of childhood (6, 7). The presence of focal pathologies does not always result in focal EEG findings and can also be multifocal or generalized (8, 9). Additionally, epilepsy surgery has been successfully utilized in patients with focal pathologies and generalized or multiregional interictal discharges (10).

The exact pathophysiology behind ESES remains poorly understood (11). Although there may be limited clinical seizure burden associated with ESES, it now is relatively well-accepted that ESES often is associated with significant cognitive regression and changes in behavioral functioning (12, 13). Cognitive changes may reflect a lack of developmental progress, global loss of skills or emerging specific deficits depending upon the localization of ESES discharges (14). Behavioral change most often is characterized by hyperactivity and increased aggression (13, 15) and may persist even with remission of ESES (16). Notably, younger age at onset of ESES may be associated with worse cognitive and behavioral outcomes (16, 17). Despite the low clinical seizure burden in many patients with ESES, resolution of this electrographic pattern has been shown to be associated with better cognitive outcomes (13, 15, 18). The resolution of cognitive decline may vary based on the etiology associated with ESES. In one study, idiopathic ESES was associated with a more favorable outcome than ESES from a symptomatic/structural etiology (19).

ESES has proven to be a refractory pattern of epileptic encephalopathy (20, 21), and so far there is no consensus on the optimal medical treatment for ESES (22). Resective surgery is an alternative to medical therapies, especially in cases of ESES associated with brain lesions. Despite the lack of robust comparative studies, the clinical and electrographic outcomes of the reported surgical series seem to outperform the outcomes of any other medical therapy (7, 23–28). A recent meta-analysis of the different treatment options showed improvement in either Electroencephalography (EEG) or cognitive functioning in response to benzodiazepines in 68% of patients; to steroids in 81%, and to surgery in 90% of patients vs. improvement in only 49% of patients in response to traditional Anti-epileptic Drugs (AEDs) (29). In most of the surgical series mentioned above, patients underwent surgery to control refractory seizures and ESES. Only rarely was surgery performed in the setting of rare or no seizures and ESES (24, 28).

In the current case series, the data support previous studies indicating efficacy of resective surgery in managing ESES by demonstrating good long-term outcomes from clinical, electrographic and neurocognitive standpoints in most patients. We expand upon the previous literature by providing the largest single center study examining resective surgery for ESES, with examination of outcomes related to the most commonly used procedure (hemispherectomy/hemispherotomy), as well as more limited resections.

## Materials and Methods

### Patients

The institutional review board at the Children's Hospital of Wisconsin approved this case series. A medical record review was completed of the electronic medical record system at the Children's Hospital of Wisconsin for patients who had a diagnosis of ESES and underwent any type of resective surgery between 2007 and 2017. Clinical variables of interest are included in Table 1.

**Table 1:** Presurgical clinical features.

**Patient**	**Handedness**	**Age at epilepsy onset**	**Age at ESES diagnosis**	**Deficits on exam**	**Duration of epilepsy**	**Seizure semiology and frequency**	**Pre-op AEDs**	**Pre-op MRI**	**Previous epilepsy surgeries**
1	Undetermined	2 years	3 years	Spastic quadriparesis, L > R	6 years	Head atonic, daily; Staring/unresponsiveness (hypomotor), daily; Generalized motor, 2/week	Felbamate, levetiracetam, topiramate, clonazepam	Diffuse volume loss, manifestation of prenatal injury, L > R	No
2	L	1st day of life	8 years	R hemiparesis	12 years	Nocturnal arousals, frequency not specified	Valproic acid, clonazepam	LMCA encephalomalacia	No
3	L	3rd day of life	1 year	None	3.5 years	Tonic spasms resolved by the age of 1 year. Undefined frequency	Phenobarbital, valproic acid	L hemimegalencephaly	No
4	L	3.5 years	8 years	R hemiparesis, R hemianopsia	2.5 years	R head version, R arm clonic, every 6–10 weeks; SGTCS, 3 altogether (status)	Valproic acid, topiramate	LMCA encephalomalacia	No
5	R	2 years	10 years	L hemiparesis; L hemianopsia	10 years	Staring and unresponsiveness, 2–3/week until age 4 years.	Felbamate	RMCA encephalomalacia	No
6	L	2 years, 11 months	10.5 years	R hemiparesis; R hemianopsia	7.5 years	R face clonic with unresponsiveness; R head version → R arm tonic → SGTS; stopped by 6 years	Valproic acid	L hemispheric volume loss; Sequelae of interventricular hemorrhage in prematurity	No
7	R	2 years	3.5 years	L hemiparesis	6 years	Non-specific aura → ictal emesis → L versive seizure, infrequent stopped by 3.5 years	Valproic acid, felbamate	Right frontoparietal encephalomalacia; Sequelae of congenital hydrocephalus	Yes, subtotal R frontal lobectomy at 6 years of age
8	L	4 years	6 years	None	4 years	Nausea → ictal emesis → dialeptic, 1–2/month; Facial flushing → L arm clonic → L head version → SGCTS, unknown frequency	Oxcarbazepine, lacosamide, clorazepate, Ketogenic diet	Unilateral right ventriculomegaly	Yes, R parietal and occipital limited cortical resections
9	L	N/A	3 years	R spastic hemiparesis	N/A	No clinical seizures	Clonazepam	LMCA encephalomalacia	No
10	L	1 year	1 year, 4 months	R spastic hemiparesis	4 months	Hypomotor → R version → bilateral asymmetric clonic sz → R arm clonic sz, with intact consciousness, 3/week	Levetiracetam, oxcarbazepine, clobazam	LMCA encephalomalacia	No
11	R	2 years	8.5 years	L spastic hemiparesis	8.5 years	Autonomic (pallor, cyanosis) → hypomotor, daily; L arm clonic → SGTCs, frequency unknown	Valproic acid, phenytoin, clobazam	R hemispheric malformation of cortical development	No
12	L	1st week of life	7.5 years	R spastic hemiparesis	8 years	R hemibody clonic sz, 1 every 1–4 weeks; Dialeptic, Daily; Atonic sz, Rare	Felbamate, phenytoin, clobazam	Diffuse L hemispheric encephalomalacia; Sequelae of interventricular hemorrhage	L temporal lobectomy; L parieto-occipital resection
13	L	3 years 10 months	5.5 years	None	2 years 8 months	R face clonic seizure, daily; R arm clonic sz → SGTCS, rare	Clobazam, lamotrigine, prednisone	Normal	No
14	L	2 years	8 years	None	7 years	GTCS, frequency not specified. Stopped by age 8 years	Levetiracetam, lamotrigine, zonisamide, clonazepam	Non-specific white matter abnormalities	No

*SGTCS, Secondarily Generalized Tonic Clonic Seizure; LMCA, left middle cerebral artery; RMCA, right middle cerebral artery; UE, upper extremity; LE, lower extremity; R, right; L, left; sz, seizure*.

Fourteen patients were identified, 7 males and 7 females. Age range at the onset of epilepsy was 1 day to 4 years. Age at diagnosis of ESES ranged from 1 to 10.5 years (median age of 6 years) with a mean delay of 4.6 years between the onset of epilepsy and recognition or development of ESES.

### Neuropsychological and Behavioral Data

All identified patients were referred for pre- and post-surgical neuropsychological evaluations. Cognitive measures used varied depending upon age and level of functioning of each patient; however, a general level of intellectual and adaptive behavioral functioning was attained for each.

Intellectual abilities were assessed using the Wechsler Intelligence Scale for Children (WISC)-V (30), WISC-IV (31), Wechsler Preschool and Primary Scales of Intelligence (WPPSI)-IV (32), Differential Abilities Scale (33)–II (34), or Mullen Scales of Early Learning (35). Adaptive skills were assessed using the Vineland Adaptive Behavior Scales (VABS) (36) or the Adaptive Behavior Assessment Scale (ABAS) (37). Attention problems were assessed via parental report on the Achenbach Child Behavior Checklist (CBCL) (38, 39). Scores on the intellectual and adaptive measures are reported as standard scores, for which normative data have a mean of 100 and standard deviation of 15. That means that scores between 85 and 115 are within the broad average range. Attention problems as assessed on the CBCL are reported as T-scores, which have a mean of 50 and standard deviation of 10, with anything above *T* = 70 considered clinically significant.

### EEG Data

Patients included in the study had preoperative EEG reports that documented the presence of ESES. Patients with EEG showing slow spike and wave consistent with other epileptic encephalopathies, such as Lennox-Gastaut Syndrome were excluded. We only included patients with the electrographic findings of ESES and any clinical phenotype of developmental regression (see section Pre-surgical Neuropsychological and Behavioral Functioning). The estimation of the interictal burden and the criteria used to determine ESES varied according to the initial clinical reader of the EEG. To more consistently determine the interictal burden, an epileptologist (AM) reviewed EEG records for each patient. Due to the variability in determining SWI combatable with ESES by different epileptologists, we included patients who had an SWI of at least 75%. Consistent with Loddenkemper (40) and Sanchez Fernandz (3), SWI was determined by counting the number of seconds occupied by at least one epileptiform discharge in the first 5 min of the first sleep cycle of the pre- and post-operative EEGs and calculating the percentage of seconds showing epileptiform discharges. SWI of wakefulness were also calculated similarly. Random 5-min portions of awake EEGs with eye blinks, posterior dominant rhythm or both were used to calculate the awake SWI and spike frequency. Additional analysis included the location and concordance of the interictal discharges with the imaging findings, non-epileptiform interictal abnormalities, presence or absence of sleep structures, and the posterior dominant rhythm (Table 2).

**Table 2:** Pre- and post-surgical EEG features.

**Patient**	**Age of diagnosis of ESES (years)**	**Pre-op SWI of NREM sleep**	**Pre-op** **SWI of wakefulness**	**Interictal location, generalized vs. focal**	**Concordance between interictal location and MRI pathology**	**Post-op** **SWI of NREM sleep**	**Post-op** **SWI of wakefulness**	**Last post-op EEG**
1	3	100%	90%	Generalized	No	5%	0%	18 months
2	8	100%	Unavailable	Focal	Yes	7%	0%	3.5 years
3	1	100%	100%	Focal	Yes	90%	80%	7 years
4	8	99%	34%	Focal	Yes	30%	13%	32 months
5	10	>85%	Unavailable	Focal	Yes	0%	0%	24 months
6	10.5	100%	Unavailable	Focal	Yes	3%	1%	4.5 years
7	3.5	91%	27%	Focal	Yes	6%	6%	2 years
8	6	88%	26%	Focal	Yes	0%	0%	18 months
9	3	98%	69%	Focal	Yes	0%	0%	14 months
10	1.3	82%	60%	Focal	Yes	20%	7%	18 months
11	8.5	79%	3%	Focal	Yes	20%	25%	2.5 years
12	7.5	100%	14%	Focal	Yes	0%	0%	9 years
13	5.5	100%	94%	Focal	N/A	84%	7%	3 years
14	8	100%	Unavailable	Focal	N/A	20%	0%	13 months

*R, right; L, left*.

### Outcome Assessment

Post-surgical functioning was characterized based on clinical neurological follow-up at least 1-year post-surgery (mean = 4.3 years). The average time from surgery to post-surgical outpatient neuropsychological follow-up was 15.75 months (range 4–46 months).

## Results

### Pre-surgical Clinical and Imaging Features

Ten patients had a baseline hemiparesis with or without hemianopsia and one had spastic quadriparesis. Semiology of clinical seizures was variable and included motor and non-motor seizures. The pre-operative clinical seizure burden ranged from 1 to 2 seizures per month to daily seizures, all of which were refractory to different combinations of AEDs. Eight out of 14 patients had refractory seizures prior to surgery and 6/14 had no seizures prior to surgery. Interestingly, five patients had frequent seizures for 4–6 years before the clinical seizure burden decreased dramatically or resolved prior to surgery. The reason for this improvement/resolution of clinical seizures is not entirely clear but could be explained by either ongoing medical therapy or the natural history of the disease itself. One patient did not have any known clinical seizures at all and ESES was found on EEG due to concerns for developmental delays and early demonstration of handedness. However, ESES burden and cognitive deficits were documented in all patients irrespective of clinical seizure burden.

Brain magnetic resonance imaging (MRI) showed structural abnormalities in 12 patients. The most common finding was encephalomalacia consistent with previous middle cerebral artery strokes (5/14). Other MRI findings included perinatal injury leading to diffuse bilateral encephalomalacia more prominent in one hemisphere (3/14), unilateral hemispheric malformation of cortical development (2/14), and congenital hydrocephalus with unilateral pathology (2/14). Imaging was totally normal or showed non-specific white matter changes in 2/14. MRI findings did not differ related to age of onset of epilepsy *F*_(4,13)_ = 1.70, *p* = ns or age at ESES diagnosis *F*_(4,14)_ = 0.198, *p* = ns. Pre-surgical clinical features are detailed in Table 1.

### Pre-surgical EEG Findings

Most patients (12/14) had pre-operative EEGs that matched the most widely used definition of ESES per Tassinari (41). Of the remaining two patients, one had SWI of 79% and the other had SWI of 82%, and both were considered to have ESES by the original EEG reader. SWI of wakefulness was also calculated and ranged from 0 to 100%. There was a positive correlation between SWI of sleep and that of wakefulness, *r*_(9)_ = 0.67, *p* = 0.05. Morphology of interictal discharges was not uniform and ranged from polyspikes to sharply contoured, rhythmic delta waves.

Only one patient had pre-surgical epileptiform interictal discharges with a morphology consistent with Benign Focal Epileptiform Discharges of Childhood (BFEDCh) (42). The presence of focal features in the setting of ESES is termed “atypical ESES,” a finding commonly seen in lesional or acquired cases of ESES (8, 43–45). All but one patient had atypical ESES with focal features. These focal features included strictly uni-focal discharges, independent multifocal but uni-hemispheric discharges, independent multifocal bi-hemispheric discharges and bi-hemispheric/generalized discharges with a lateralized maximum. Only one patient had generalized discharges with no clear laterality or focality. The location of at least one of the epileptiform foci always matched the location of the MRI pathology when present. Non-epileptiform interictal abnormalities, such as focal or background slowing were found in 13/14 patients. Only one patient had normal EEG background and she had the lowest sleep SWI (79%). Sleep structures were absent in 3/14 patients. Table 2 details the pre-operative EEG findings.

### Pre-surgical Neuropsychological and Behavioral Functioning

Based on the sample mean, intellectual abilities at the pre-surgical assessment were impaired. Most (8/14; 57%) had intellectual abilities in the range of intellectual disability (i.e., FSIQ <70). Parental ratings of adaptive functioning were generally commensurate and mildly impaired. Parent report of behavior indicated clinically significant attention problems (see Tables 3, 4). SWI was not related to intellectual functioning or attention problems; however, of the portion of the sample for which adaptive functioning was available, a higher SWI percentage during sleep was associated with lower adaptive functioning *r*_(10)_ = −0.683, *p* = 0.03. That is, the higher SWI during sleep in the context of ESES, the lower the patient's daily functioning. In 42.9% of the sample, the results of the pre-surgical neuropsychological evaluation were thought to reflect a plateau or decline in cognitive functioning relative to prior neuropsychological evaluations. This pre-surgical impression of cognitive functioning was not related to pre-surgical SWI in sleep [SWI% for plateau/decline *M* = 94.5%, *SD* = 8.31; for stable *M* = 96.3, *SD* = 6.37; *F*_(1, 13)_ = 0.192, ns] Those patients with plateau or decline had longer duration of epilepsy before surgery [*M* = 8.9 years (*SD* = 2.24)] than those whose cognitive development continued to progress [*M* = 5.1 years (*SD* = 3.08)], *F*_(1, 13)_ = 5.56, *p* = 0.038.

**Table 3:** Group means of intellectual, adaptive and behavioral data.

	**Pre-surgical assessment**		**Post-surgical assessment**	
	**Mean (SD)**	**Range (min–max)**	**Mean (SD)**	**Range (min–max)**
Age at evaluation (years)	7.56 (2.61)	3.3–12.3	9.11 (2.64)	6.6–15.4
Intellectual abilities	67.57 (15.37)	42.0–96.0	61.79 (14.57)	38.0–87.0
Adaptive skills	71.72 (14.03)	39.0–95.0	71.11 (12.32)	40.0–82.0
Attention problems	70.91 (12.90)	57–97	67.50 (7.29)	57–83

*See description of measures used in text*.

**Table 4:** Individual pre- and post-surgical intellectual, adaptive and behavioral data.

	**Pre-surgical data**				**Post-surgical data**				
**Patient**	**Impression of functional change**	**FSIQ (SS)**	**Adaptive functioning (SS)**	**Attention problems (T-scores)**	**Time since surgery for follow-up (months)**	**FSIQ (SS)**	**Adaptive functioning (SS)**	**Attention problems (T-scores)**	**Impression of functional outcome**
1	Stable	42	39	–	5	38	40	83	Positive
2	Decline	53	67	66		54	–	–	Positive
3	Stable	58	67	80	38	50	–	–	Decline
4	Decline	79	79	62	8	85	77	57	Positive
5	Stable	80	–	97	10	80	–	66	Positive
6	Stable	45	69	59	46	45	69	66	Positive
7	Decline	75	85	71	28	63	79	71	Decline
8	Stable	73	77	90	4	58	75	73	Decline
9	Decline	72	70	–	28	70	73	75	Positive
10	No Data	49	66	62	20	59	70	59	Positive
11	Decline	58	95	69	6	63	82	69	Positive
12	Decline	66	75	–	6	51	74	61	Decline
13	Stable	96	–	57	7	87	–	66	Decline
14	Stable	58	–	67	11	62	–	64	Positive

*Stable indicates continued appropriate development. Decline indicates loss of skills or lack of developmental progress. Positive outcome was defined as arrest of decline, gains in performance or stability of function*.

### Post-surgical Seizure Outcomes and Pathology

Mean age at time of surgery was 7.9 years (range 3.5–12 years). The most common surgical procedure was hemispherectomy (primarily hemispherotomies) in 10/14 patients (six left; four right). The following procedures were performed in one patient each: subtotal frontal lobectomy sparing the primary motor cortex, temporo-parieto-occipital disconnection, parieto-occipital resection and limited frontal corticectomy. Three patients had received prior resective surgeries to treat epilepsy and ESES, including limited parieto-occipital cortical resections, subtotal frontal lobectomy and temporal lobectomy. Of these, two patients ended up receiving a hemispherectomy and one a temporo- parieto-occipital disconnection.

Pathological studies revealed reactive gliosis as the most common finding 7/14 followed by focal cortical dysplasia in 5/14. One patient had cortical dysplasia and mesial temporal sclerosis and one had mesial temporal sclerosis only. All patients who did not receive a hemispherectomy underwent invasive monitoring prior to surgery. No new neurological deficits developed after surgery in 13/14 patients. Only one patient developed a new right hemiparesis post-operatively after left hemispherotomy.

Post-surgical clinical neurological follow up ranged from 1.5 to 11 years (mean 4.3 years). We used the ILAE seizure outcome scale (46). At the time of last clinical follow-up, 10 patients were seizure free (ILAE 1), two had significantly reduced seizure burden (ILAE 3), and 1 had no change in seizure burden (ILAE 5). Altogether, 12/13 patients had favorable seizure outcome. One patient never had known clinical seizures, therefore clinical seizure outcome did not apply. Hemispherectomy was best associated with seizure freedom *X*_2_(2, *n* = 12) = 8.00, *p* = 0.018. All patients that received hemispherotomy were seizure free, and 1 patient that received a temporo- parieto-occipital disconnection was also seizure-free. Two patients continued to have seizures but with a significant improvement following surgery, one received a parieto-occipital resection and the other frontal lobectomy. No improvement in seizure burden was seen in one patient who received a limited frontal corticectomy. Six patients were completely weaned off all AEDs. One patient remained on a small dose of valproic acid for mood stabilization. The remainder remained on AEDs post-surgically.

Whether epilepsy was refractory or not preoperatively did not have an independent impact on seizure outcomes (seizure free, reduced seizure, no change) *X*_2_(2, *n* = 12) = 0.80, *p* = ns. Pathology was also not related to seizure outcome *X*_2_(6, *n* = 12) = 0.91, *p* = ns. Table 5 details the post-operative clinical features and seizure outcomes.

**Table 5:** Post-surgical outcomes.

**Patient**	**Age at surgery (years)**	**Type of surgery**	**Extra-operative invasive monitoring**	**Pathology**	**Follow-up duration**	**AEDs**	**New deficits**	**ILAE seizure outcome**	**Neuropsychological outcome**
1	6	R parieto-occipital resection	Yes	Focal cortical dysplasia	2	Felbamate, topiramate	None	III	Positive
2	12	L hemispherotomy	No	Gliosis	3.5	None	None	I	Positive
3	3.5	L hemispherotomy	No	Focal cortical dysplasia	7	None	R hemiparesis	I	Decline
4	6	L hemispherotomy	No	Focal cortical dysplasia	7.5	None	None	I	Positive
5	12	R hemispherotomy	No	Focal cortical dysplasia	3.5	None	None	I	Positive
6	10.5	L hemispherotomy	No	Hippocampal sclerosis; Focal cortical dysplasia	4	None	None	I	Positive
7	8	R hemispherotomy	No	Hippocampal sclerosis	2	Valproic acid, for behavioral concerns	None	I	Decline
8	8	R temporo-parieto-occipital disconnection	Yes	Gliosis	2	Oxcarbazepine, lacosamide	None	I	Decline
9	5	L hemispherotomy	No	Gliosis		Clonazepam	None	N/A	Positive
10	5.8	L hemispherotomy	No	Gliosis	1.5	Oxcarbazepine	None	I	Positive
11	10.5	R hemispherotomy	No	Focal cortical dysplasia	2.5	Clobazam	None	I	Positive
12	8	L hemispherotomy	No	Gliosis	9	Valproic acid, felbamate	None	I	Decline
13	6.5	L corticectomy in lateral superior frontal gyrus	Yes	Gliosis	3.5	Phenytoin, lamotrigine, clobazam	None	V	Decline
14	9	L frontal lobectomy	Yes	Gliosis	11	Lamotrigine	None	III	Positive

*Positive is defined as arrest of decline, gains in performance or stability of function. R, right; L, left*.

### Post-surgical EEG Findings

Timing of follow up EEG ranged from 12 months to 9 years post-operatively (mean 3 years). ESES resolved (with a SWI of 0–30%) in 12/14 patients, all of whom achieved good seizure outcomes. There was no relation between type of surgery (hemispherectomy vs. other) and ESES resolution; *X*_2_(1, *n* = 14) = 0.525, *p* = ns. Of 2 patients who did not have resolution of ESES, one received a limited frontal cortical resection (no improvement in seizure burden) and the other a left hemispherectomy (clinical seizure freedom). This last patient had discrepant clinical and EEG outcomes, with seizure freedom but persistent ESES. The location of the persistent ESES remained in the operated hemisphere without change.

Posterior dominant rhythm was absent pre-operatively but returned in one patient. Sleep structures (vertex waves and spindles) were absent pre-operatively and returned in two patients.

Table 2 details the post-op EEG findings.

### Post-surgical Neuropsychological and Behavioral Outcomes

All patients completed post-surgical neuropsychological evaluations anywhere from 4 to 48 months post-surgery (*M* = 17.46 months; *SD* = 14.67 months). There was no significant change in the mean level of intellectual functioning, *F*_(1,12)_ = 1.53, *p* = 0.24, or adaptive functioning, *F*_(1, 7)_ = 1.42, *P* = 0.27, of the sample following surgery. There was no significant change in mean level attention problems, *F*_(1, 8)_ = 1.35, *p* = 0.28. Despite the lack of statistically significant changes, it was notable that at the post-surgical evaluation, only 27% of patients had clinically significant attention problems (see Table 2). However, pre-surgical impression of functioning was not related to post-surgical outcome *X*_2_(1, *n* = 13) = 0.008, *p* = ns. The overall stability of neuropsychological and behavioral functioning in this sample is important as it reflects a resolution of the previously identified decline or plateauing of functions prior to surgery. This is particularly meaningful as standardized scores are based on comparison an individual's performance to that of same-aged typically developing peers. Thus, stability in standardized scores indicates that those patients who previously demonstrated regression or plateau in development returned to a more typical rate of development post-operatively in order to maintain the same comparison to peers.

Nine of the 14 patients (64%) were considered to have a positive cognitive outcome, clinically defined as arrest of previously identified cognitive decline, gains in intellectual or adaptive skill performance or stability of pre-surgical functioning. Of the five patients who demonstrated a decline in functioning post-surgically, 2/5 showed mild decline in intellectual abilities (<0.5 standard deviation) and three showed moderate decline (~1 standard deviation). Two of these five also demonstrated persistent ESES (Table 5).

Stability in post-surgical intellectual abilities was not associated with resolution of ESES *X*_2_(1, *n* = 13) = 0.430, *p* = ns; type of surgery (hemispherectomy vs. other) *X*^2^(1, *n* = 13) = 0.410, *p* = ns or change in seizure burden *X*_2_(2, *n* = 13) = 0.709, *p* = ns. Adaptive functioning post-surgery was available only from patients with resolved ESES and 88% had stable adaptive functioning. Stability in adaptive functioning was not associated with surgery type (hemispherectomy vs. other) *X*_2_(1, *n* = 8) = 0.381, *p* = ns or change in seizure burden *X*_2_(1, *n* = 8) = 0.163, *p* = ns. Post-surgical SWI was not related to adaptive functioning *r*_(8)_ = 0.145, *p* = ns. This reflects the restricted range associated with the resolved ESES.

### Case Illustration—Patient #9

**Figure 1:**
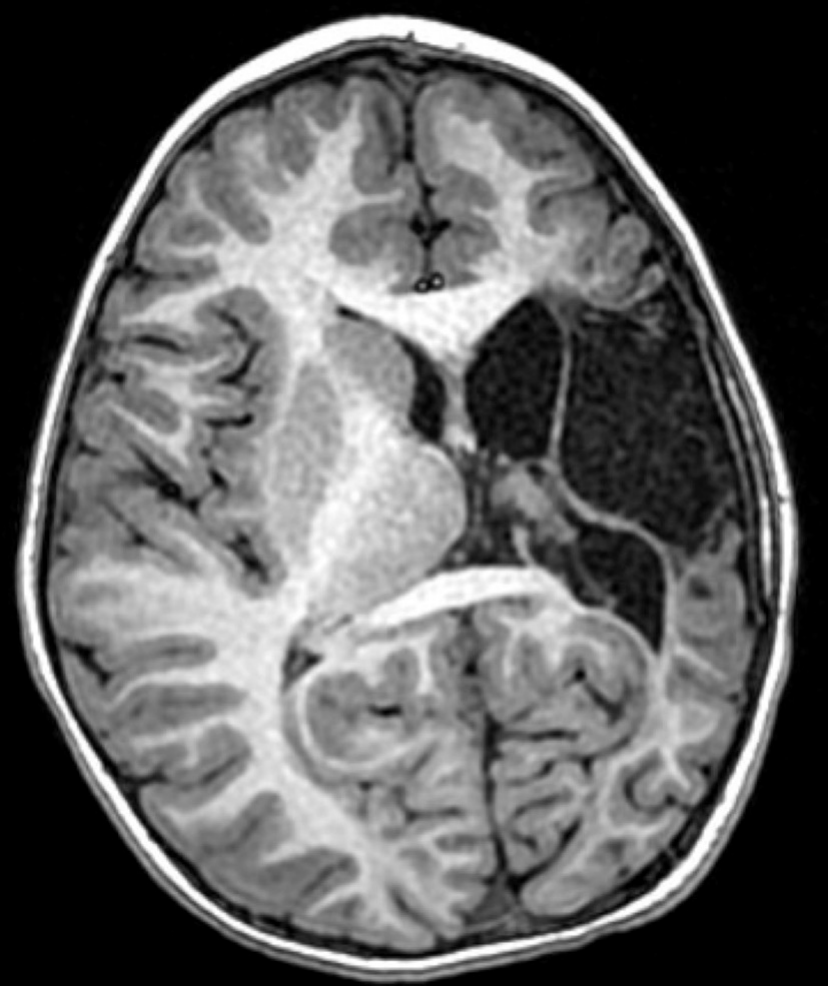
Patient #x. Axial T1-weighted MRI obtained at age 5 years, demonstrating a left middle cerebral artery distribution infarct and a disproportionately small left cerebral hemisphere.

Informed consent was obtained from the parents agreeing for this case illustration. The patient was the product of an uncomplicated pregnancy, born via c-section due to failure to progress after failed vacuum delivery. At 1 month of age, he was noted to have right sided weakness and imaging ultimately revealed a left middle cerebral artery distribution perinatal stroke (Figure 1). Workup revealed Factor V Leiden and prothrombin mutations.

He demonstrated mild developmental delays in infancy and received Botox injections to manage right-sided spasticity. Language development was delayed but he did begin to speak in phrases by age 4 years. By age 5 years it was apparent that he was not gaining new words/skills.

**Figure 2:**
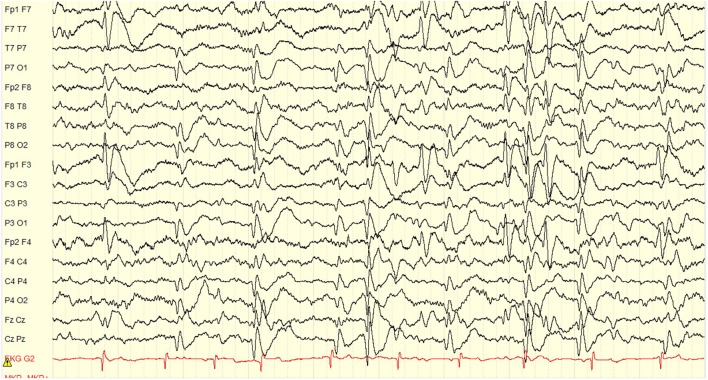
Pre-operative EEG showing an ESES pattern occupying 90% of the sleep recording. The interictal discharges localize to the left frontal, and left occipital regions independently. Volume conduction to the right frontal and right occipital regions is also seen, but the maximum clearly lateralizes to the left hemisphere. Display setup: bipolar double banana montage, 10 s page, low frequency filter 1 Hz, high frequency filter 70 Hz, gain 100 microvolts per cm.

Paroxysmal spells suspicious for seizures began around 18 months of age in retrospect with episodic brief episodes of shuddering. By age 3 years he was having staring spells. Scalp EEG at the time demonstrated independent left frontal and occipital interictal discharges with a spike wave index (SWI) of 90–100% meeting criteria for ESES on the left (Figure 2). At age 4 years he was having rare staring spells and frequent nocturnal awakenings, neither of which were ever captured or shown to represent clinical seizures. Thus, the patient was considered not to have had any proven clinical seizures. By the age of 4.5 years, he was felt to not be retaining newly taught information and had regressed with toilet training. He completed a pre-surgical neuropsychological evaluation at age 5 years, 9 months. Comparison of results with prior assessment reflected minimal developmental progress over the last 7 months. He demonstrated a mild discrepancy between low average verbal intellectual abilities and mildly deficient non-verbal or visual-spatial abilities. His memory skills were intact, but he demonstrated significant attention problems and attained a diagnosis of Attention Deficit/Hyperactivity Disorder (ADHD). On physical exam he demonstrated a dense right hemiplegia with a circumduction gait and left visual field defect (complete homonymous hemianopsia could not be confirmed due to age). By the age of 4.5 years he was felt to not be retaining newly taught information and had regressed with toilet training. He case was presented in the multidisciplinary epilepsy conference after which his family was offered a left hemispherotomy in the hopes of optimizing his development and reducing the risk of future seizures.

**Figure 3:**
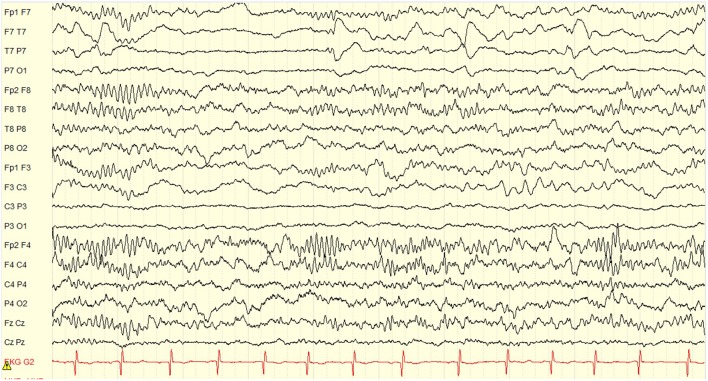
Post-operative EEG obtained 14 months after left hemispherotomy. There is complete resolution of ESES with no interictal discharges. Only expected non-epileptiform changes of continuous slow and asymmetry of sleep structures in the left hemisphere are seen. Display setup: bipolar double banana montage, 10 s page, low frequency filter 1 Hz, high frequency filter 70 Hz, gain 100 microvolts per cm.

At the age of 5 years and 10 months he underwent an uneventful lateral hemispherotomy. He remained free of clinical seizures after surgery. Within 3 months of surgery he was once again toilet-trained but continued to have difficulties with attention, hyperactivity and obsessive behaviors. Post-operative EEG showed complete resolution of ESES (Figure 3). At last clinical follow-up, 2.5 years after surgery (age 8 years) he was off seizure medication, taking only guanfacine for ADHD, and making excellent progress in school. He completed a post-surgical neuropsychological evaluation at age 8 years, 3 months; 28 months after surgery. Whereas, pre-surgically he demonstrated minimal developmental progress, in the post-surgical evaluation he demonstrated significant gains in verbal abilities which improved to the average range. His non-verbal or visual spatial abilities remained mildly deficient, consistent with what is often seen as a result of early dominant/left hemisphere injury and subsequent functional reorganization. Memory remained intact, but attention problems persisted and remained consistent with ADHD.

## Discussion

### Clinical Outcomes

Our case series includes 14 patients, the largest single center cohort of patients to have undergone resective surgery for ESES. Similar to other reports, most patients received hemispherotomy surgery (10/14) (23, 25–28, 47). The seizure outcomes for the hemispherotomy subgroup are universally excellent with seizure freedom achieved in all patients. One other patient achieved seizure freedom following a temporo-parieto-occipital disconnection. Our results are consistent with the available literature for outcomes related to hemispherotomy. Our data also adds to the limited prior report of patients who responded favorably to limited resective surgeries including temporal lobectomy (24), temporo-parieto-occipital resection (23), and frontal lobectomy (28).

Long term seizure freedom in the setting of ESES can be achieved despite the variable etiologies and surgical procedures utilized in the setting of ESES. Eight patients with >4-years follow-up had excellent seizure outcome, seven of which remained seizure free. This suggests that excellent clinical seizure outcome (whether freedom from or substantial improvement in seizure frequency) can persist for years and is not an immediate or transient effect. Additionally, all three patients who had previous surgeries achieved seizure freedom at last follow up. We conclude that a failed previous surgery should not preclude further surgical evaluation in case of ESES or seizure persistence.

Although patients with ESES are known to have low seizure frequency, most patients undergoing surgery described in the literature had a high seizure burden, sometimes daily (23, 25–27) and only few had infrequent or no seizures for all or part of the ESES course (24, 28). This is the first study to report a high number of patients (6/14) with rare or no clinical seizures responding to surgery from an electrographic and neuropsychological standpoints. We suggest that clinical seizure freedom, even if extended for years, should not be considered a contraindication to resective surgery for ESES causing cognitive and/or behavioral decline.

This series also demonstrates the long-term effect of surgery on the EEG with seven patients continuing to show resolution of ESES beyond 2 years. This prolonged EEG improvement has been rarely reported before (24, 25) as most previous studies had EEG follow up <2 years post-operatively or did not elaborate on the length of the follow up (23, 26, 27).

The presence of bilateral MRI abnormalities has been previously found to correlate with a less favorable outcome (23). However, we had two patients with bilateral MRI abnormalities who did well post-operatively, one achieving prolonged seizure freedom and the other having seizures only with missed medications.

From an electrographic standpoint, it is well-known that there is an overlap between atypical benign focal epilepsies of childhood and ESES (7, 11). These patients tend to have normal brain imaging and no focal deficits on exam. The only patient in our cohort with BFEDCh had no focal deficits and did not respond to a limited frontal cortical resection. It is possible that there is a correlation between the morphology of interictals and presence of abnormal brain imaging. However, this statement is based on a single patient observation who underwent a limited resection. Further studies are needed before making any conclusion about the relationship between interictal morphology and surgery for ESES.

The distribution of interictal discharges in patients with ESES and structural lesions is variable, ranging from generalized (23, 26) to focal ESES (25, 28). Our findings were compatible with the reported literature with three patients showing either generalized or bifrontal maxima, and 11 showing focal ESES. Interestingly, we had one patient with right and left independent interictal foci who underwent a right temporo-parieto-occipital disconnection and remained seizure free at the last follow up 1.5 years later. The presence of an independent focus contralateral to the location of the lesion did not affect the outcome. Although conclusions cannot be made based on this one individual, it is plausible to suggest that the presence of bilateral independent interictal foci is not necessarily a poor prognostic factor for surgery, especially if one focus is concordant with the location of the MRI lesion.

### Neuropsychological Outcomes

Although there was no statistically significant change in the mean intellectual or adaptive functioning, most of the sample demonstrated stability in functioning and was determined clinically to demonstrate positive outcome. This stability in functioning supports the hypothesis that eliminating ESES allows for more positive developmental outcomes (26). As noted previously, stability in standardized scores indicates that post-operatively patients returned to a more typical rate of development in order to maintain a steady comparison of their performance to that of same-aged typically developing peers. The absence of statistically significant changes in standardized scores on formal measures does not preclude good outcome as any significant change in scores would have necessitated patients demonstrate cognitive developmental progress at a much greater rate than is observed in typical development. Within this sample, there was no statistical relation between the stability in intellectual and adaptive functioning and ESES or clinical seizure burden post-surgery; however, these analyses were limited by power and lack of variability in intellectual and adaptive outcomes as most patients showed stability in outcomes. On an individual patient level, nine of the 14 patients with post-surgical evaluation data demonstrated positive neuropsychological outcomes. Of the five patients who demonstrated declines in functioning, two demonstrated persistent ESES.

### Limitations

Like previous studies addressing epilepsy surgery for ESES, our study has limitations in the lack of a comparison group and use of a retrospective design. Although this is the largest cohort reported on to date, 14 remains a small sample size. The limited sample is mainly due to the rarity of ESES cases that are eligible for resective surgery. Another limitation is the single hospital study environment with expected practice and possible patient selection bias. A prospective, multicenter study comparing patients who qualify for resective surgery vs. another group treated medically is needed to obtain high level evidence on the efficacy of variable treatments for ESES.

## Conclusion

Resective surgery can be an effective treatment in managing ESES resulting in excellent seizure control and stabilization of neurocognitive outcomes. Typical patients are those who suffered a perinatal insult to the brain with a clear unilateral lesion on brain imaging. However, bilateral imaging abnormalities or normal brain anatomy are not always contraindications, and surgery should be considered in carefully selected patients. Hemispherectomy, temporo-occipito-parietal resection/disconnection and frontal lobectomy are among the procedures utilized in this cohort. Limited cortical resection may not be as effective as bigger resections, especially without a clear MRI lesion. Excellent long-term clinical outcomes of seizure control, resolution of ESES and neurocognitive status stability are observed years after surgery. This case series adds data from the largest, single center cohort of patients related to the long-term effects of different resective surgery procedures in the treatment of ESES.

## Data Availability Statement

All datasets generated for this study are included in the article/supplementary material.

## Ethics Statement

The studies involving human participants were reviewed and approved by The institutional review board at the Children's Hospital of Wisconsin. Written informed consent to participate in this study was provided by the participants' legal guardian/next of kin. Written informed consent was obtained from the patient's next of kin for the publication of this case study.

## Author Contributions

AM: study concept and design, literature search, data collection and interpretation, drafting and critical revision of manuscript. JK and ML: literature search, data collection, data analysis and interpretation, and critical revision of manuscript. Y-WL: data collection. SL: critical revision of manuscript.

### Conflict of Interest

The authors declare that the research was conducted in the absence of any commercial or financial relationships that could be construed as a potential conflict of interest.
